# A hyper-resolving polynomial aperture and its application in microscopy

**DOI:** 10.1186/s43088-022-00209-z

**Published:** 2022-02-14

**Authors:** A. M. Hamed

**Affiliations:** Physics Department, Faculty of Science, Ain Shams University, Cairo, Egypt

**Keywords:** Polynomial aperture, Point spread function, Siemen’s star pattern, Confocal scanning laser microscope

## Abstract

**Background:**

A hyper-resolving aperture composed of a polynomial distribution is suggested. The point spread function (PSF) is computed and compared with that corresponding to linear, quadratic, and circular apertures. In addition, the influence of the number of zones on the PSF is discussed. An application on confocal scanning laser microscope using Siemen’s star pattern as an object considering the polynomial apertures is given.

**Results:**

We have made polynomial apertures using MATLAB code, and we tested the resolution from the computation of the cut-off spatial frequency obtained from the computation of the point spread function.

**Conclusions:**

We get compromised resolution and contrast for the polynomial apertures as compared with uniform circular apertures.

## Background

The microscope used in the processing called CSLM is mainly composed of two objective lenses arranged in tandem and having common short focus where the scanned object is placed. Coherent illumination of the microscope is provided by a laser beam and a coherent point detector is placed in the imaging plane. This confocal microscope is studied by many authors [1–10]. An explanation for the imaging of confocal microscopy attaining super-resolution in confocal imaging was presented [7].

It was shown early [3] that resolution has been improved by using annular aperture as compared with the open circular aperture. The microscope resolution is basically dependent on the wavelength of illumination and the numerical aperture NA or the aperture size for certain focal length, hence the theoretical limit of resolution is computed as follows: resolution = *λ*/NA. While the distribution in the aperture has a little effect on the resolution and contrast as in [11–17].

The main object of the proposed methods of modulation is based on improving the transverse resolution of the confocal microscope outlined previously in many publications [18–24]. Recently, resolution and contrast measurements of optical microscope based on PSF engineering is investigated in [25–27] while the resolution and contrast enhancement in laser scanning microscopy using dark beam is discussed in [28]. A scanning twice in confocal microscopy for better resolution is studied in [29] and confocal microscopy with pinhole super-resolution is discussed in [30]. The relation between the optical transfer function and the PSF using obstructed apertures is widely used to compare the performance of different optical systems in [31]. Enhancing the performance of fluorescence emission difference microscopy using beam modulation is given in [32], while the effects of polarization on the deexcitation dark focal spot in STED microscopy is discussed in [33]. Recent publication in aperture modulation, using annular Hermite Gaussian aperture, is investigated in [34].

In this study, the motivation for choosing the hyper-resolving apertures which has the form of polynomial distribution is discussed showing further improvement in resolution compared with the open circular aperture.

## Methods

In the first model, we proposed five equal zones of higher-order polynomial *ρ*^8^ at the center ending with a linear function of *ρ* at the surface of the aperture as follows: *ρ*^8^, *ρ*^6^, *ρ*^4^, *ρ*^2^, and *ρ*.

The selection of five zones is presented to fulfill the arrangement assumed for the polynomial.

For the second model, this number is doubled since the center is assumed dark. In general, we can take any number of zones either even or odd depending on the proposed distribution. Hence, the first model has odd number of zones *N* = 5, while the second model has *N* = 10.

**Fig. 1:**
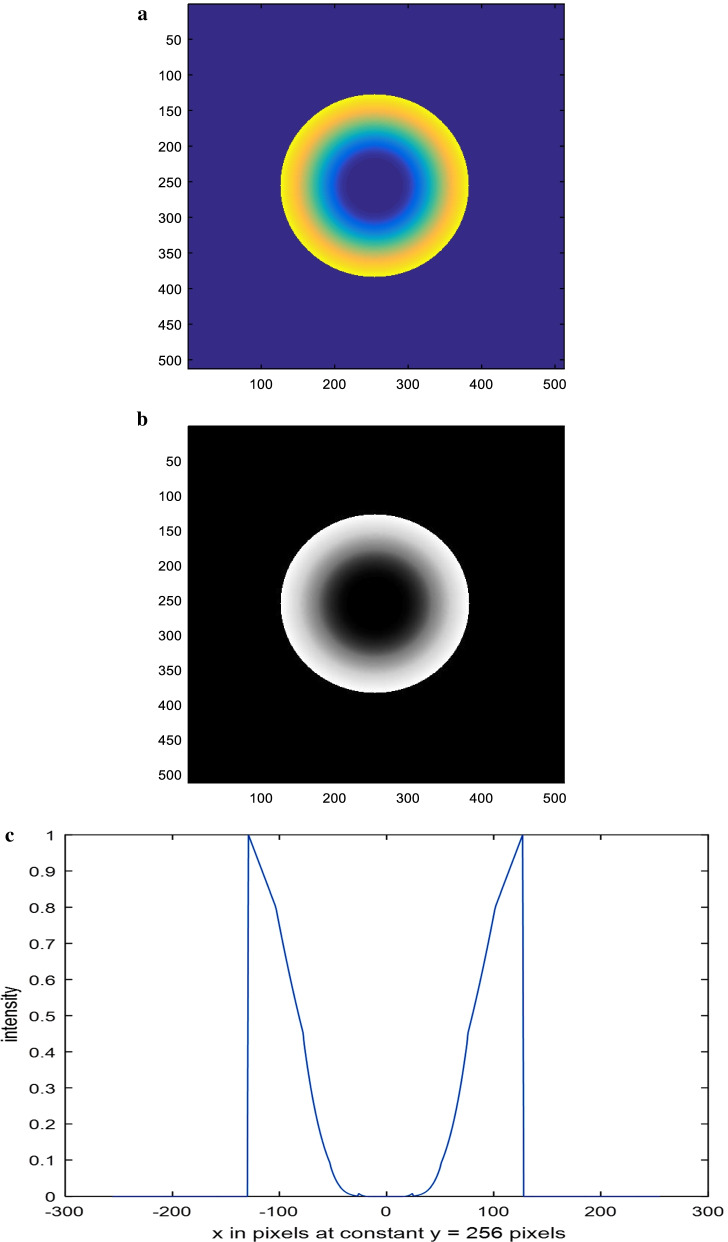
**a** Color image corresponding to the first model of polynomial aperture showing the five concentric layers. **b** Gray-scale image of a circular aperture in the form of a polynomial distribution with five equal zones. The concentric zones have distributions *ρ*^8^, *ρ*^6^, *ρ*^4^, *ρ*^2^ and *ρ* computed from the aperture center. The matrix dimensions have 512 × 512 pixels and the total radius of the aperture = 128 pixels. **c** The intensity plot of the polynomial aperture shown in the (**b**) at the center of the aperture at constant *y* = 256 pixels

The assumed polynomial aperture has five equal zones of distributions, starting from the center, represented as *ρ*^8^, *ρ*^6^, *ρ*^4^, *ρ*^2^, and *ρ* as shown in Fig. 1. The corresponding line plot is shown as in Fig. 1c. In our case, the central zone has transmission intensity proportional to *ρ*^8^ instead of zero for the annular aperture.

Now, the polynomial aperture is written as follows:

1 $$\begin{aligned} P\left( \rho \right) & = a\rho ^{8 } , \quad {\text{for}}\; 0 \le \rho < 0.2\rho _{\max } \\ & = b \rho^{6 } , \quad {\text{for}}\; 0.2 \le \rho _{\max } < 0.4\rho _{\max } \\ & = c\rho ^{4 } , \quad {\text{for}}\; 0.4 \le \rho_{\max } < 0.6 \rho_{\max } \\ & = d \rho^{2 } , \quad {\text{for}}\; 0.6 \le \rho _{\max } < 0.8\rho _{\max } \\ & = e\rho , \quad {\text{for}}\; 0.8 \le \rho _{\max } < \rho _{\max } \\ \end{aligned}$$

*a*, *b*, *c*, *d*, *e*, constants are proportional to the cross-sectional areas of the corresponding zones.

In this model, referring to Eq. (1),

$$a = 0.04 \pi \rho_{\max }^{2} ,\;\; b = 0.12\pi \rho_{\max }^{2} ,\;\; c = 0.20 \pi \rho_{\max }^{2} ,\;\;d = 0.28\pi \rho_{\max }^{2} , \;\;e = 0.36\pi \rho_{\max }^{2}$$

Hence, $a + b + c + d + e = \pi \rho_{\max }^{2}$ is the total area of open circular aperture of radius $\rho_{\max }$.

*ρ* = (*u*, *v*) is the radial coordinate corresponding to the Cartesian coordinates (*u*, *v*) and $\rho_{\max }$ is the total aperture radius.

The PSF corresponding to the polynomial aperture, described in Equ 1, is computed by operating the Fourier transform upon Eq. (1) considering coherent illumination emitted from spatially filtered Laser beam. Hence, the PSF is represented in integral form in polar coordinates as follows:

2 $$h\left( {r;\theta } \right) = \mathop \smallint \limits_{0}^{{\rho_{\max } }} \mathop \smallint \limits_{0}^{2\pi } P\left( \rho \right)\exp \left[ { - \frac{j2\pi }{{\lambda f}}\rho r\cos \left( {\Phi - \theta } \right)} \right] \rho {\text{d}}\rho {\text{d}} \Phi$$

where *u* = *ρ* cos *Φ*, *v* = *ρ* sin *Φ* are the Cartesian coordinates in the aperture plane corresponding to the polar coordinates (*ρ*, Φ), while *x* = *r* cos *θ*, *y* = *r* sin *θ* are the Cartesian coordinates in the Fourier or focal plane corresponding to the polar coordinates (*r*, *θ*). The Fourier transform lens has focal length = f.

Since the aperture has circular symmetry of revolution, equation, (2) is reduced to a function of r only as follows [11]:

3 $$h_{{\text{model 1}}} \left( r \right) = 2\pi \mathop \smallint \limits_{0}^{{\rho_{\max } }} P\left( \rho \right) J_{0} \left( {\frac{2\pi }{{\lambda f}}\rho r} \right) \rho {\text{d}}\rho$$

where $J_{0} \left( x \right)$ represents the Bessel function of zero order and the Bessel function of any order n $J_{n} \left( x \right)$ is represented by the following summation:

$$J_{n} \left( x \right) = \mathop \sum \limits_{m = 0}^{\infty } \frac{{\left( { - 1} \right)^{m} }}{{m!\left( {m + n} \right)!}} \left( \frac{x}{2} \right)^{n + 2m} .$$

Substituting Eq. 1 in Equ 3, we get:

4 $$\begin{aligned} h_{{\text{model 1}}} \left( r \right) & = 2 \pi \left\{ {a \mathop \smallint \limits_{0}^{{0.2\rho_{\max } }} \rho^{8} J_{0} \left( {\frac{2\pi }{{\lambda f}} \rho r} \right) \rho {\text{d}}\rho + b\mathop \smallint \limits_{{0.2\rho_{\max } }}^{{0.4\rho_{\max } }} \rho^{6} J_{0} \left( {\frac{2\pi }{{\lambda f}}\rho r} \right) \rho {\text{d}}\rho } \right. \\ & \quad + c\mathop \smallint \limits_{{0.4\rho_{\max } }}^{{0.6\rho_{\max } }} \rho^{4} J_{0} \left( {\frac{2\pi }{{\lambda f}} \rho r} \right) \rho {\text{d}}\rho + d\mathop \smallint \limits_{{0.6\rho_{\max } }}^{{0.8\rho_{\max } }} \rho^{2} J_{0} \left( {\frac{2\pi }{{\lambda f}} \rho r} \right) \rho {\text{d}}\rho \\ & \quad \left. { + e \mathop \smallint \limits_{{0.8\rho_{\max } }}^{{\rho_{\max } }} \rho J_{0} \left( {\frac{2\pi }{{\lambda f}} \rho r} \right) \rho {\text{d}}\rho } \right\} \\ \end{aligned}$$

Solving Eq. (4), we finally get the corresponding result for the PSF as follows:

5 $$\begin{aligned} h_{{\text{model 1}}} \left( r \right) & = \frac{{J_{1} \left( {W_{5} } \right)}}{{W_{5} }} - 0.08\mathop \sum \limits_{i = 1}^{4} \frac{{J_{1} \left( {W_{i} } \right)}}{{W_{i} }} + 0.4 \frac{{J_{2} \left( {W_{1} } \right)}}{{W_{1}^{2} }} + 0.08 \frac{{J_{2} \left( {W_{2} } \right)}}{{W_{2}^{2} }} - 0.24 \frac{{J_{2} \left( {W_{3} } \right)}}{{W_{3}^{2} }} \\ & \quad - 0.56 \frac{{J_{2} \left( {W_{4} } \right)}}{{W_{4}^{2} }} + 0.36\left( { \frac{{J_{0} \left( {W_{5} } \right)}}{{W_{5}^{2} }} - \frac{{J_{0} \left( {W_{4} } \right)}}{{W_{4}^{2} }}} \right) - 0.96 \frac{{J_{3} \left( {W_{1} } \right)}}{{W_{1}^{3} }} + 1.28\frac{{J_{3} \left( {W_{2} } \right)}}{{W_{2}^{3} }} \\ & \quad + 1.6\frac{{J_{3} \left( {W_{3} } \right)}}{{W_{3}^{3} }} + 0.72 \mathop \sum \limits_{i = 1}^{N} \left( {\frac{{J_{i} \left( {W_{4} } \right)}}{{W_{4}^{3} }} - \frac{{J_{i} \left( {W_{5} } \right)}}{{W_{5}^{3} }} } \right) - 1.92 \frac{{J_{4} \left( {W_{1} } \right)}}{{W_{1}^{4} }} - 5.76\frac{{J_{4} \left( {W_{2} } \right)}}{{W_{2}^{4} }} \\ & \quad + 15.36 \frac{{J_{5} \left( {W_{1} } \right)}}{{W_{1}^{5} }} \\ \end{aligned}$$

where *i* = (1, 3, 5, …, *N*), $$W_{1} = \frac{2}{f} \left( {0.2\rho_{\max } } \right)r$$, $$W_{2} = \frac{2}{f} \left( {0.4\rho_{\max } } \right)r$$,

$$W_{3} = \frac{2}{f} \left( {0.6\rho_{\max } } \right)r$$, $$W_{4} = \frac{2}{f} \left( {0.8\rho_{\max } } \right)r$$, $$W_{5} = \frac{2}{f} \left( {\rho_{\max } } \right)r$$.

The PSF corresponding to the second model is computed by following the above analysis; Eq. (4) except the integral limits changed following the new intervals between the ten concentric equal zones of different distributions. Hence, we write the PSF as follows:

6 $$\begin{aligned} h_{{\text{model 2}}} \left( r \right) & = 2\pi \left\{ {a\mathop \smallint \limits_{{0.1\rho_{\max } }}^{{0.2\rho_{\max } }} \rho^{8 } J_{0} \left( {\frac{2\pi }{{\lambda f}} \rho r} \right) \rho {\text{d}}\rho + b \mathop \smallint \limits_{{0.3\rho_{\max } }}^{{0.4\rho_{\max } }} \rho^{6 } J_{0} \left( {\frac{2\pi }{{\lambda f}} \rho r} \right)\rho {\text{d}}\rho } \right. \\ & \quad + c\mathop \smallint \limits_{{0.5_{\max } }}^{{0.6_{\max } }} \rho^{4 } J_{0} \left( {\frac{2\pi }{{\lambda f}} \rho r} \right) \rho {\text{d}}\rho + d\mathop \smallint \limits_{{0.7\rho_{\max } }}^{{0.8\rho_{\max } }} \rho^{2 } J_{0} \left( {\frac{2\pi }{{\lambda f}} \rho r} \right) \rho {\text{d}}\rho \\ & \quad \left. { + e\mathop \smallint \limits_{{0.9\rho_{\max } }}^{{\rho_{\max } }} \rho J_{0} \left( {\frac{2\pi }{{\lambda f}}\rho r} \right) \rho {\text{d}}\rho } \right\} \\ \end{aligned}$$

It is noted that the other five integrals are set equal to zero for the dark zones in the (B/W_polynomial_) aperture. The cross-sectional areas corresponding to the transparent zones have the values:

$$a = 0.03 \pi \rho_{\max }^{2} , \;\;b = 0.07\pi \rho_{\max }^{2} ,\;\; c = 0.11\pi \rho_{\max }^{2} ,\;\;d = 0.15\pi \rho_{\max }^{2} ,\;\; e = 0.19\pi \rho_{\max }^{2} .$$

We finally get the PSF corresponding to the second model of polynomial aperture as follows:

7 $$\begin{aligned} h_{{\text{model 2}}} \left( r \right) & = 0.19 \left[ { \frac{{J_{0} \left( {W_{10} } \right)}}{{W_{10}^{2} }} - \frac{{J_{0} \left( {W_{9} } \right)}}{{W_{9}^{2} }}} \right] - 0.38\mathop \sum \limits_{i = 1,3,5, \ldots } \left[ { \frac{{J_{i} \left( {W_{10} } \right)}}{{W_{10}^{3} }} - \frac{{J_{i} \left( {W_{9} } \right)}}{{W_{9}^{3} }}} \right] \\ & \quad + \mathop \sum \limits_{i = 1}^{5} \left[ { \frac{{J_{1} \left( {W_{2i} } \right)}}{{W_{2i} }} - \frac{{J_{1} \left( {W_{2i - 1} } \right)}}{{W_{2i - 1} }}} \right] - 0.24 \left[ {\frac{{J_{2} \left( {W_{2} } \right)}}{{W_{2}^{2} }} - \frac{{J_{2} \left( {W_{1} } \right)}}{{W_{1}^{2} }}} \right] \\ & \quad - 0.42 \left[ {\frac{{J_{2} \left( {W_{4} } \right)}}{{W_{4}^{2} }} - \frac{{J_{2} \left( {W_{3} } \right)}}{{W_{3}^{2} }}} \right] - 0.44 \left[ {\frac{{J_{2} \left( {W_{6} } \right)}}{{W_{6}^{2} }} - \frac{{J_{2} \left( {W_{5} } \right)}}{{W_{5}^{2} }}} \right] \\ & \quad - 0.3 \left[ {\frac{{J_{2} \left( {W_{8} } \right)}}{{W_{8}^{2} }} - \frac{{J_{2} \left( {W_{7} } \right)}}{{W_{7}^{2} }}} \right] + 1.44\left[ { \frac{{J_{3} \left( {W_{2} } \right)}}{{W_{2}^{3} }} - \frac{{J_{3} \left( {W_{1} } \right)}}{{W_{1}^{3} }}} \right] + 1.68\left[ { \frac{{J_{3} \left( {W_{4} } \right)}}{{W_{4}^{3} }} - \frac{{J_{3} \left( {W_{3} } \right)}}{{W_{3}^{3} }}} \right] \\ & \quad + 0.88\left[ { \frac{{J_{3} \left( {W_{6} } \right)}}{{W_{6}^{3} }} - \frac{{J_{3} \left( {W_{5} } \right)}}{{W_{5}^{3} }}} \right] - 5.76\left[ { \frac{{J_{4} \left( {W_{2} } \right)}}{{W_{2}^{4} }} - \frac{{J_{4} \left( {W_{1} } \right)}}{{W_{1}^{4} }}} \right] \\ & \quad - 3.36\left[ { \frac{{J_{4} \left( {W_{4} } \right)}}{{W_{4}^{4} }} - \frac{{J_{4} \left( {W_{3} } \right)}}{{W_{3}^{4} }}} \right] + 11.52\left[ {\frac{{J_{5} \left( {W_{2} } \right)}}{{W_{2}^{5} }} - \frac{{J_{5} \left( {W_{1} } \right)}}{{W_{1}^{5} }}} \right] \\ \end{aligned}$$

An application in microscopy is given, particularly in the case of the CSLM [1–5], provided with polynomial apertures of type 1 or type 2 described above, and the obtained image is computed from Eq. (8), where the polynomial aperture for both microscope objectives is given in Eq. (1) for the first model:

8 $$I\left( {x,y} \right) = \left| {\iint\limits_{ - \infty }^{\infty } {h_{{{\text{polynomial}}}} \left( {x,y} \right) \cdot h_{{{\text{polynomial}}}} \left( {x,y} \right) \cdot g\left( {x - x^{\prime } ,y - y^{\prime } } \right) {\text{d}}x^{\prime } {\text{d}}y^{\prime } } } \right|^{2}$$

Consequently, the formed image is the modulus square of the convolution product of the resultant point spread function and the complex amplitude of the object. It is written symbolically as:

$$I\left( {x,y} \right) = \left| { h_{r} \left( {x,y} \right) \otimes g\left( {x,y} \right) } \right|^{2}$$

${\text{h}}_{r} \left( {x,y} \right) = [h_{{{\text{polynomial}}}} \left( {x,y} \right)]^{2} .$; for two symmetric objectives of polynomial apertures.

Here, $h_{{{\text{polynomial}}}} \left( r \right)$ is computed from Eq. (5) for the first model and computed from Eq. (7) for the second model. The image used in the processing is the Siemen’s test chart.

For a point object, the above convolution is reduced to the resultant PSF squared computed as follows:

$$\begin{aligned} I\left( {x,y} \right) & = \left| {\iint\limits_{ - \infty }^{\infty } {h_{{{\text{polynomial}}}} \left( {x,y} \right) \cdot h_{{{\text{polynomial}}}} \left( {x,y} \right) \cdot \delta \left( {x - x^{\prime } ,y - y^{\prime } } \right) {\text{d}}x^{\prime } {\text{d}}y^{\prime } } } \right|^{2} \\ & = \left| { h^{2}_{{{\text{polynomial}}}} \left( {x,y} \right) } \right|^{2} = \left[ { \frac{{2J_{1} \left( {W_{5} } \right)}}{{W_{5} }}} \right]^{4} \\ \end{aligned}$$

when the polynomial aperture is replaced by open circular aperture [1].

## Results

A color image showing the five concentric layers is shown in Fig. 1a, while a gray-scale image of a circular aperture in the form of a polynomial distribution with five equal zones is shown in Fig. 1b. The concentric zones have distributions *ρ*^8^, *ρ*^6^, *ρ*^4^, *ρ*^2^ and *ρ* computed from the aperture center. The matrix dimensions have 512 × 512 pixels and the total radius of the aperture = 128 pixels. The intensity plot of the polynomial aperture shown in Fig. 1b at the center of the aperture at constant *y* = 256 pixels is given in Fig. 1c.

**Fig. 2:**
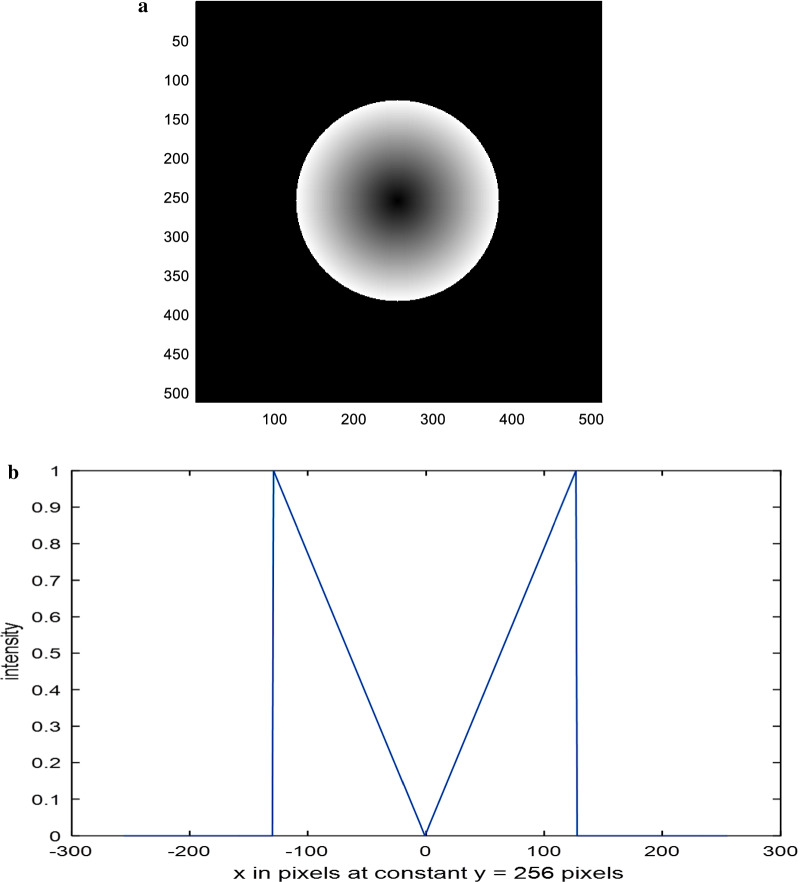
**a** An image of a linearly distributed aperture. The matrix dimensions have 512 × 512 pixels and the total radius of the aperture = 128 pixels. **b** The intensity plot of the linearly distributed aperture shown in the (**a**) at the center of the aperture at constant *y* = 256 pixels

**Fig. 3:**
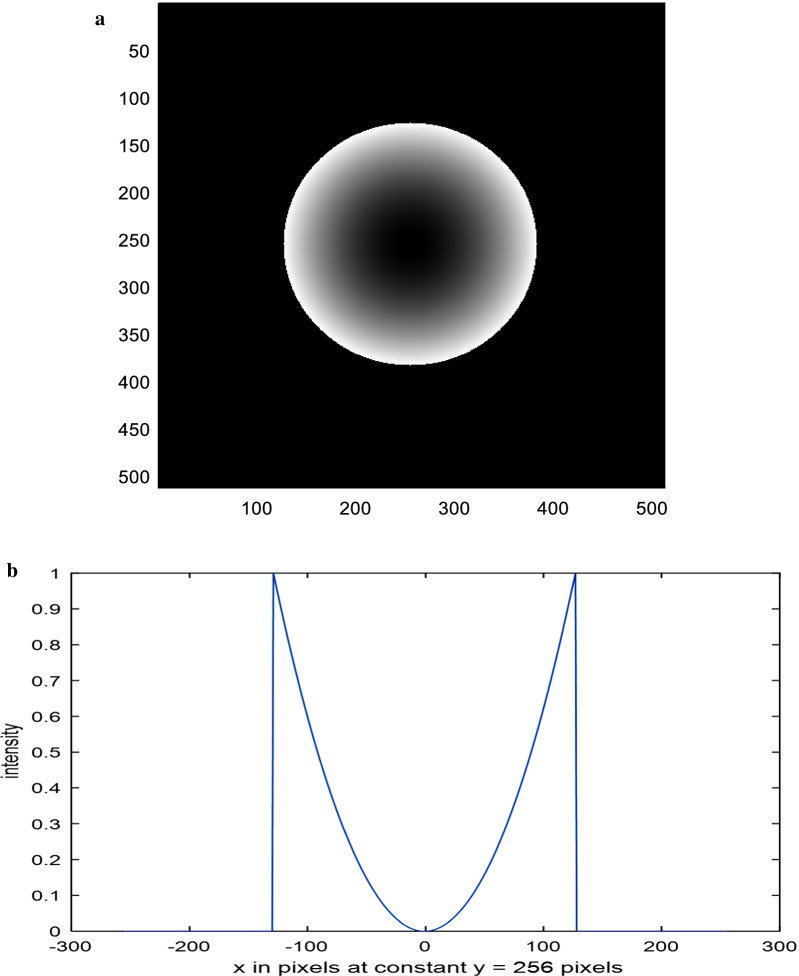
**a** An image of a quadratic distributed aperture. The matrix dimensions have 512 × 512 pixels and the total radius of the aperture = 128 pixels. **b** The intensity plot of the quadratic distributed aperture shown in the (**a**) at the center of the aperture at constant *y* = 256 pixels

**Fig. 4:**
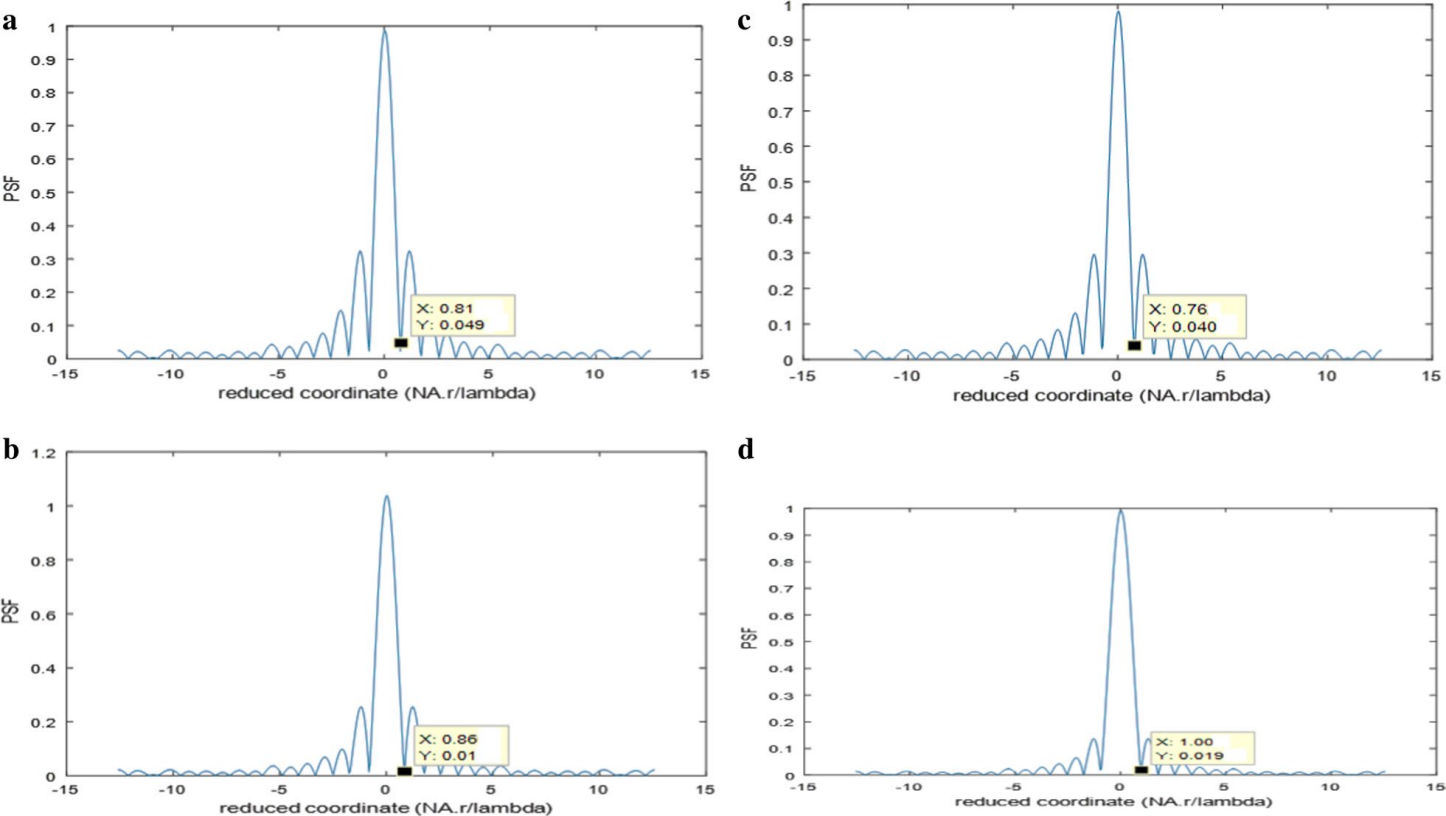
**a** Normalized PSF for the first model of polynomial aperture using FFT technique. The total diameter = 32 pixels, and the cut-off spatial frequency is located at *W*_cut-off_ = 0.81. **b** The normalized PSF for the linear aperture using FFT technique. The diameter = 32 pixels, and the cut-off spatial frequency is located at *W*_cut-off_ = 0.86. **c** The normalized PSF for the quadratic aperture using FFT technique. The diameter = 32 pixels, and the cut-off spatial frequency is located at *W*_cut-off_ = 0.76. **d** The normalized PSF for the uniform circular aperture using FFT technique. The diameter = 32 pixels, and the cut-off spatial frequency is located at *W*_cut-off_ = 1.0

The linear and quadratic apertures and their plots are shown in Figs. 2 and 3 for the sake of comparison. The normalized PSF computed by operating the FFT upon the polynomial aperture of total diameter = 32 pixels is shown in Fig. 4a. The cut-off spatial frequency is located at *W*_cut-off_ = 0.81. The comparative normalized PSF for the linear aperture of diameter = 32 pixels is shown in Fig. 4b. It is shown that the cut-off spatial frequency is located at *W*_cut-off_ = 0.86, while the normalized PSF corresponding to the quadratic aperture is shown in Fig. 4c. Improved cut-off spatial frequency is located at *W*_cut-off_ = 0.76. The comparison with the circular aperture gives greater cut-off spatial frequency at *W*_cut-off_ = 1.0 as shown in Fig. 4d.

**Fig. 5:**
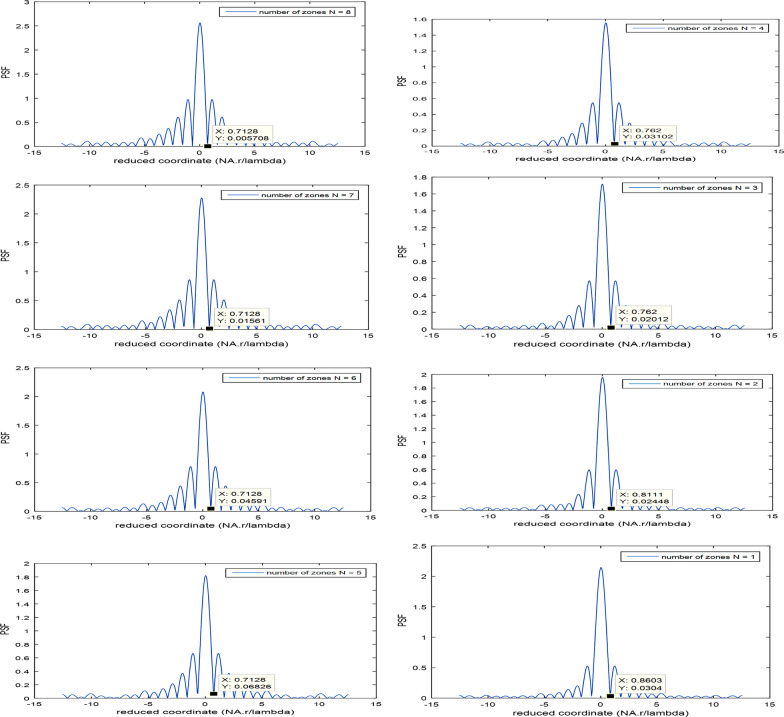
Influence of the number of zones corresponding to the first model of polynomial aperture upon the PSF. The cut-off spatial value in reduced coordinate is varied from 0.7128 for *N* = 8 up to 0.8603 for *N* = 1 which has linear distribution. It is shown the same cut-off value at 0.7128 for *N* = 5 up to *N* = 8. In addition, another equal value is shown at 0.762 for *N* = 3 and *N* = 4, while two different values are obtained for *N* = 2 at 0.8111 and *N* = 1 at 0.8603

The influence of the number of zones upon the PSF is investigated, and the PSF plots are shown in Fig. 5a–h.

**Fig. 6:**
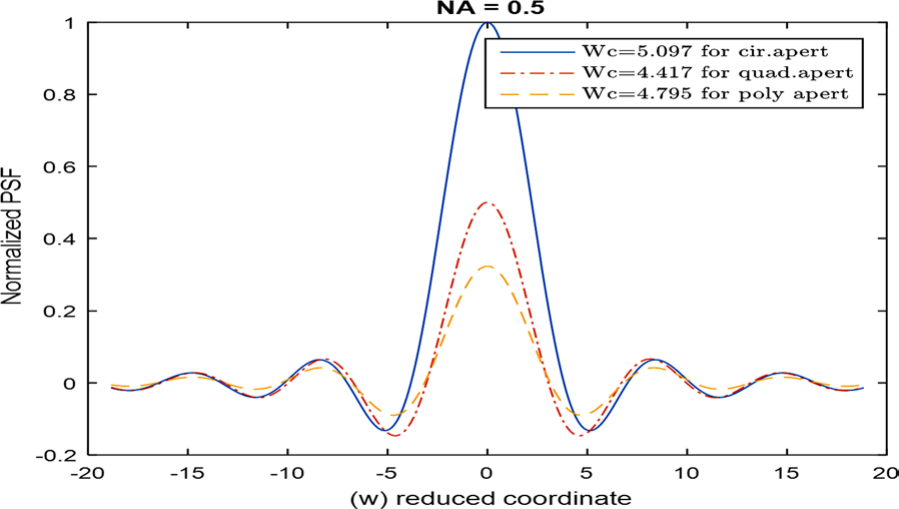
PSF corresponding to the polynomial aperture (first model) and compared with the corresponding PSF of circular and quadratic apertures using the analytical solution given by Eq. (5)

The PSF corresponding to the first model using the analytical solution represented by Eq. (5) is plotted in Fig. 6 and compared with uniform circular aperture. In the computation, it is assumed that *λ* = 500 nm and the NA = 0.5.

**Fig. 7:**
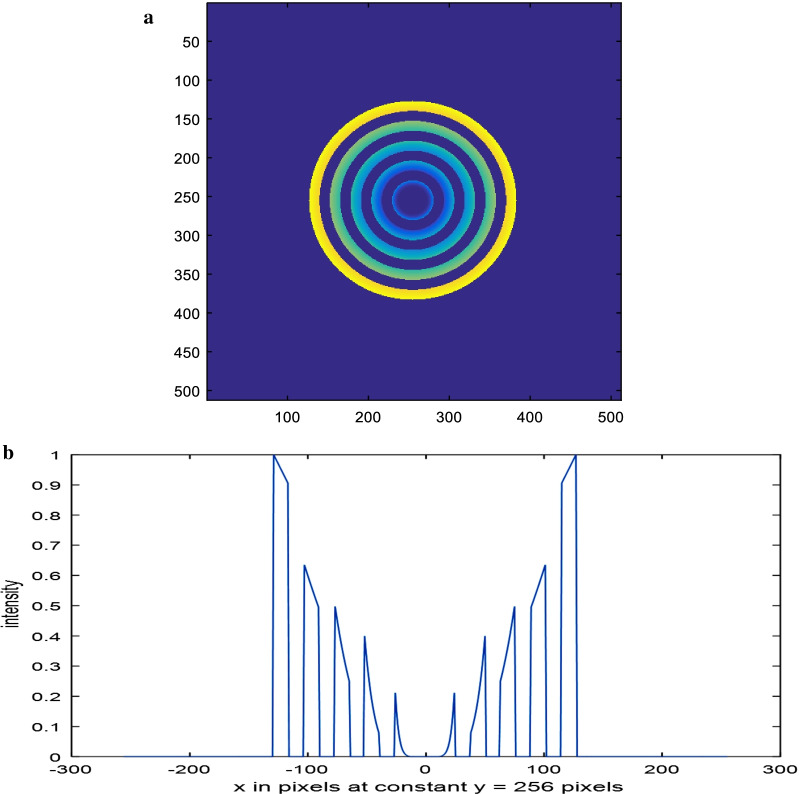
**a** Color image corresponding to the second model of polynomial aperture showing ten concentric layers of B/W_polynomial_ distribution where the center is dark. The layers from the center are 0, *ρ*^8^, 0, *ρ*^6^, 0, *ρ*^2^, 0, *ρ*. **b** The intensity plot of the second model of polynomial aperture shown in the (**a**) at the center of the aperture at constant *y* = 256 pixels

**Fig. 8:**
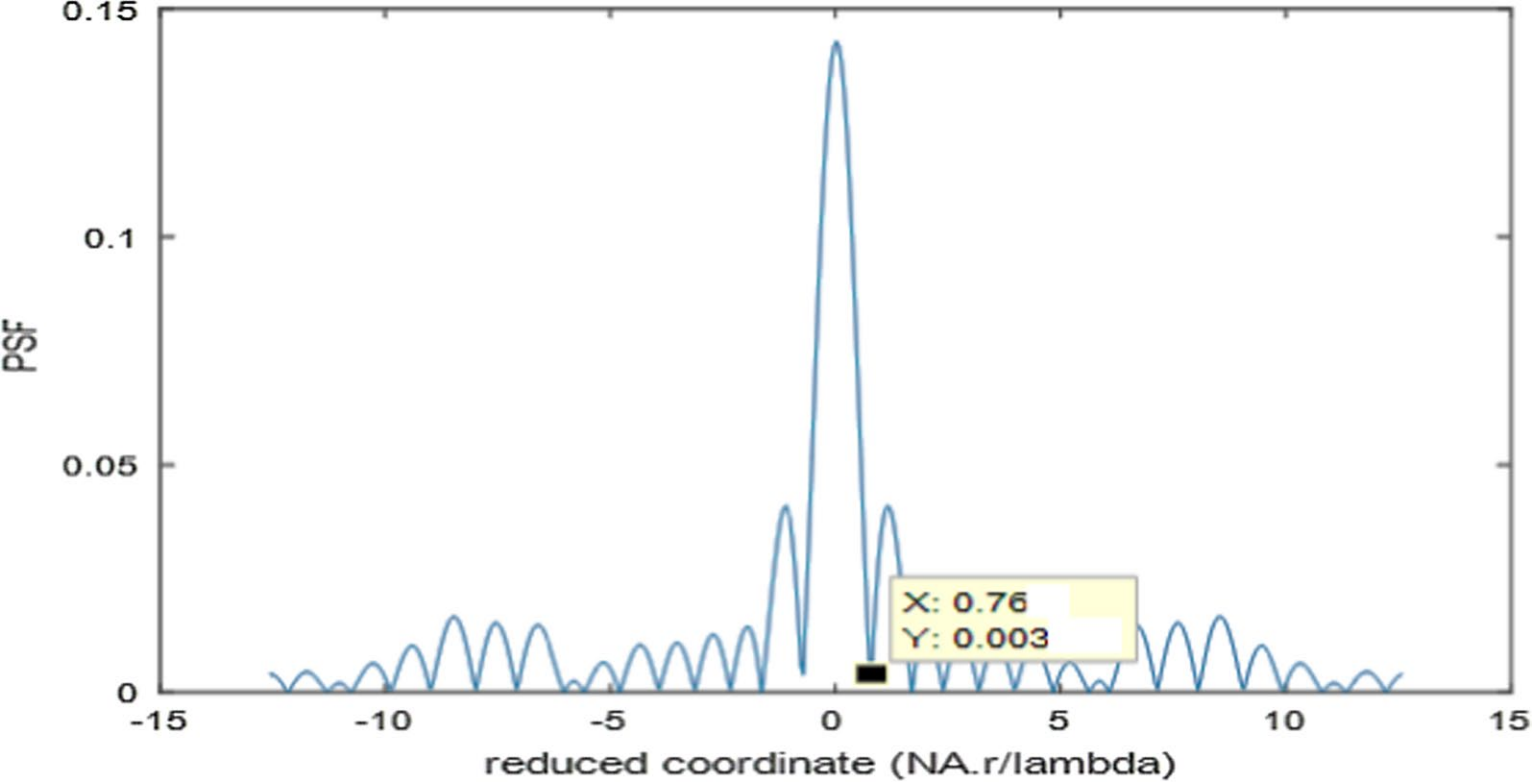
Normalized PSF for the second model of polynomial aperture using FFT technique. The total diameter = 32 pixels, and the cut-off spatial frequency is located at *W*_cut-off_ = 0.76

Color image corresponding to the second model of polynomial aperture showing ten concentric layers of B/W_polynomial_ distribution where the center is dark. The layers from the center are 0, *ρ*^8^, 0, *ρ*^6^, 0, *ρ*^4^, 0, *ρ*^2^, 0, *ρ* as shown in Fig. 7a. Its line plot is shown in Fig. 7b. The PSF corresponding to the second model of aperture using FFT technique is represented in Fig. 8.

**Fig. 9:**
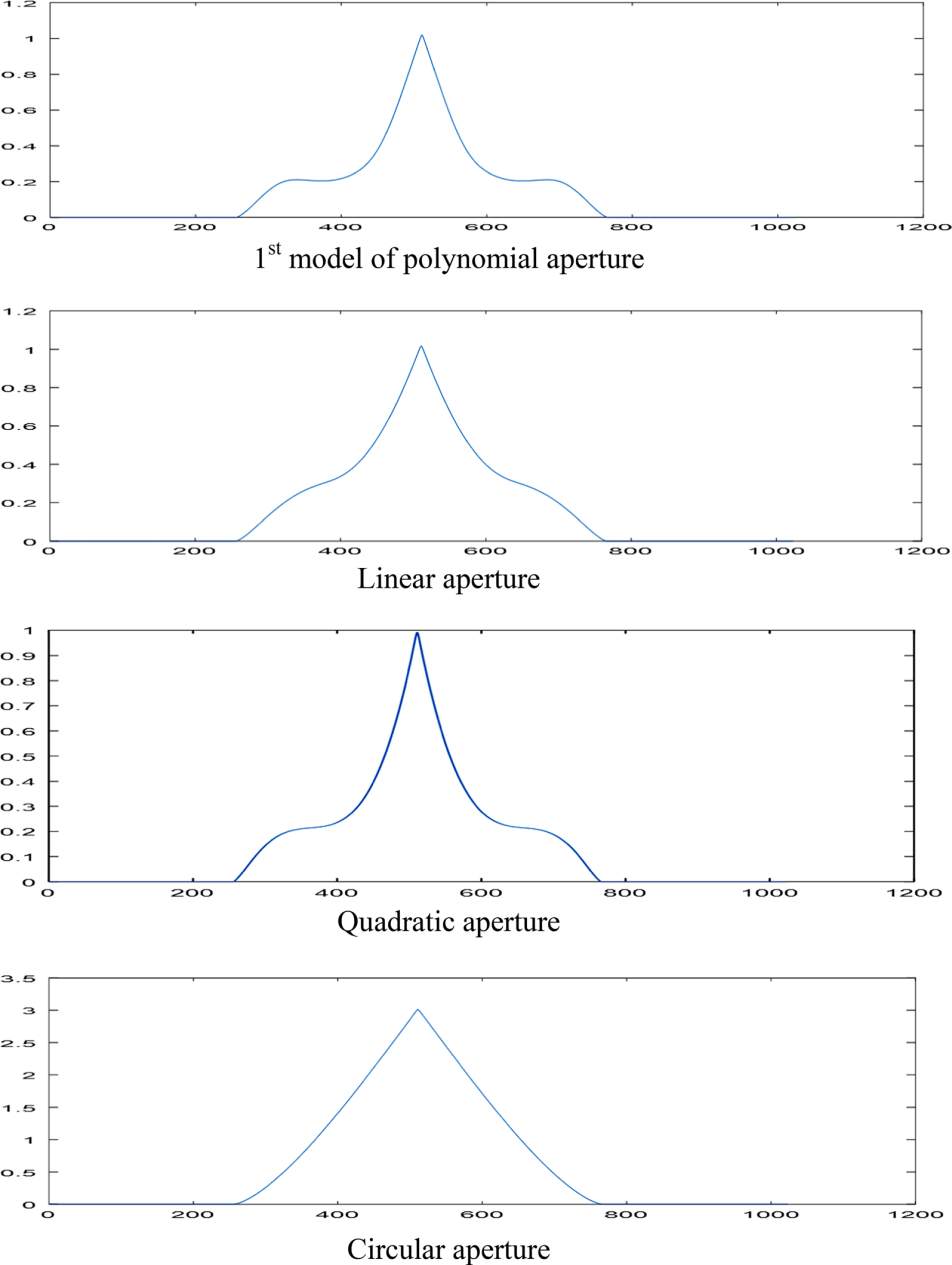
Normalized autocorrelation for the first model of polynomial, linear, quadratic, and circular apertures

The normalized autocorrelation curves for the first and second model of polynomial aperture and compared with the linear, and circular apertures are plotted in Fig. 9.

**Fig. 10:**
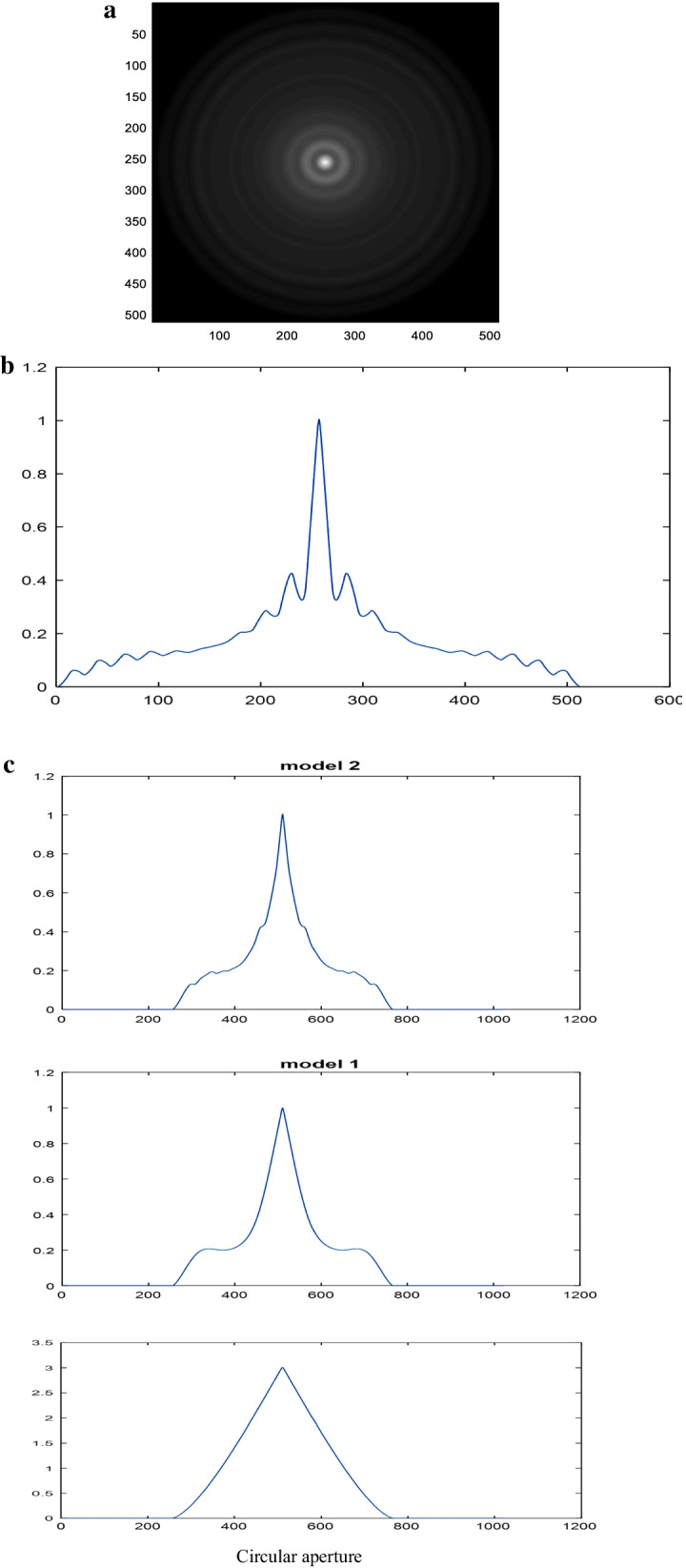
**a** The image of the autocorrelation corresponding to the polynomial aperture of the second model or the coherent transfer function (CTF) in the CSLM. **b** The autocorrelation profile corresponding to the second model of polynomial aperture computed from the FFT technique. **c** Normalized autocorrelation for the first and second models of polynomial aperture compared with the circular aperture. It is computed from the direct autocorrelation of the aperture

The image of the autocorrelation corresponding to the polynomial aperture of the second model or the coherent transfer function (CTF) in the CSLM is shown in Fig. 10a. The autocorrelation profile corresponding to the second model of polynomial aperture computed from the FFT technique is shown in Fig. 10b, where again the total band width = two times the aperture diameter = 2 × 256 = 512 pixels.

## Discussions

It is shown, from the numerical results using the FFT technique, that the polynomial aperture gives PSF curve of spatial frequency cut-off better than that obtained in case of the uniform circular and linear apertures as shown in Fig. 4.

$$W_{{\text{cut-off}}} = \, 0.81\left( {{\text{polynomial}}} \right) \, < \, W_{{\text{cut-off}}} = \, 0.86 \, \left( {{\text{linear}}} \right) \, < \, W_{{\text{cut-off}}} = \, 1.0 \, \left( {{\text{circular}}} \right).$$

While the pure quadratic aperture has better resolution compared with the polynomial aperture since *W*_cut-off_ = 0.81(polynomial) > *W*_cut-off_ = 0.76 (quadratic). It is shown that the polynomial aperture gives more intensity than the pure quadratic aperture hence compromising of resolution and contrast is attained for the polynomial aperture as compared with the linear and circular aperture.

In Fig. 5a–h, the cut-off spatial value in reduced coordinate is varied from 0.7128 for *N* = 8 up to 0.8603 for *N* = 1 which has linear distribution. It is shown the same cut-off value at 0.7128 for *N* = 5 up to *N* = 8. In addition, another equal value is shown at 0.762 for *N* = 3 and *N* = 4. While two different values are obtained for *N* = 2 at 0.8111 and *N* = 1 at 0.8603. Hence, the resolution is improved for *N* ≥ 5 as compared with the resolution for linear aperture since PSF cut-off = 0.7128 for *N* ≥ 5, while the cut-off for linear aperture is 0.8603 for one zone. The values corresponding to the cut-off plots are shown in Table 1.

**Table 1:** Influence of the number of zones *N* upon the PSF for constant NA = 0.5, and *λ*

Number of zones *N*	PSF cut-off
*N* = 1, for *P* (*ρ*)	0.8603
*N* = 2, for *P* (*ρ*, *ρ*^2^)	0.8111
*N* = 3, for *P* (*ρ*, *ρ*^2^, *ρ*^4^)	0.762
*N* = 4, for *P* (*ρ*, *ρ*^2^, *ρ*^4^, *ρ*^6^)	0.762
*N* = 5, for *P* (*ρ*, *ρ*^2^, *ρ*^4^, *ρ*^6^, *ρ*^8^)	0.7128
*N* = 6, for *P* (*ρ*, *ρ*^2^, *ρ*^4^, *ρ*^6^, *ρ*^8^, *ρ*^10^)	0.7128
*N* = 7, for *P* (*ρ*, *ρ*^2^, *ρ*^4^, *ρ*^6^, *ρ*^8^, *ρ*^10^, *ρ*^12^)	0.7128
*N* = 8, for *P* (*ρ*, *ρ*^2^, *ρ*^4^, *ρ*^6^, *ρ*^8^, *ρ*^10^, *ρ*^12^, *ρ*^14^)	0.7128

It is shown, referring to Fig. 6, that:

$$W_{{\text{cut-off}}} = \, 4.417\left( {{\text{quadratic}}} \right) \, < \, W_{{\text{cut-off}}} = \, 4.795\left( {{\text{polynomial}}} \right) \, < \, W_{{\text{cut-off}}} = \, 5.097 \, \left( {{\text{circular}}} \right)$$

in agreement with the shown numerical results using the FFT technique except the range is different depending on *λ* and NA.

The cut-off spatial frequency is located at *W*_cut-off_ = 0.76 as shown in Fig. 8. Hence, further improvement of resolution is attained as compared with the first model, linear, and circular resolutions, while it has equal resolution like quadratic aperture.

It is shown referring to all the apertures shown in Fig. 9 that the autocorrelation band width = 512 pixels. It is two times the aperture diameter as well-known. In addition, the curves are different compared with the autocorrelation of the circular aperture.

**Fig. 11:**
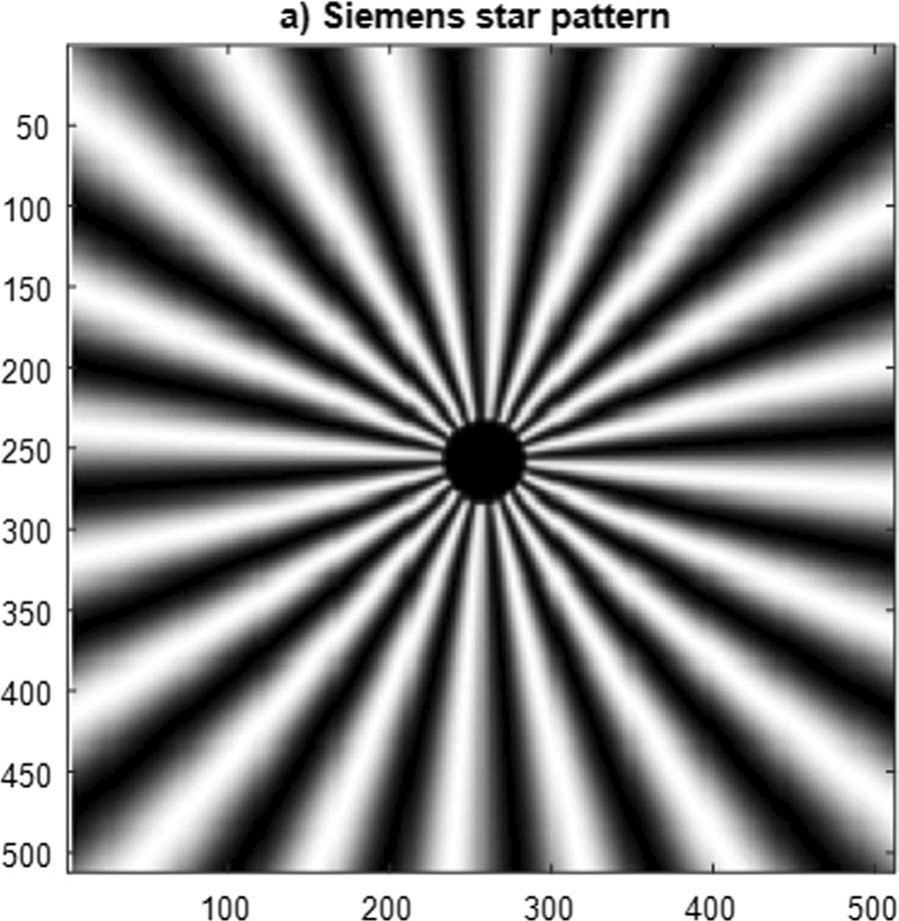
Siemen’s star pattern (a) of dimensions 512 × 512 pixels used as an object in the CSLM provided with the second model of polynomial aperture

**Fig. 12:**
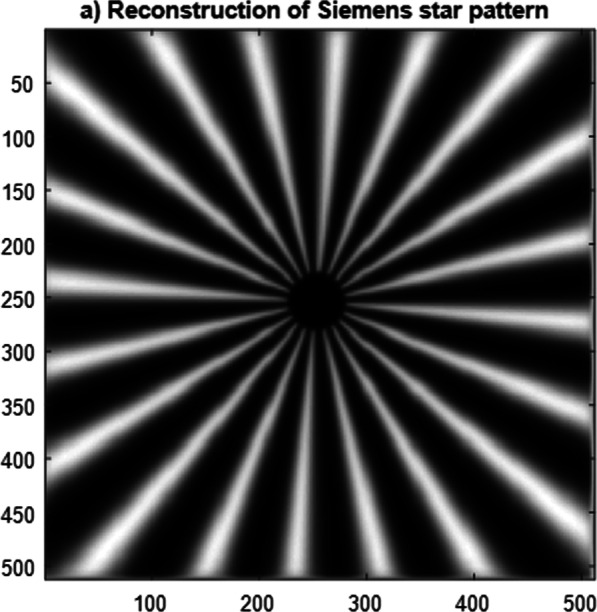
Reconstructed images using the CSLM provided with the second model of polynomial aperture

The Siemen’s star pattern of dimensions 512 × 512 pixels used as an object in the CSLM provided with the second model of polynomial aperture is given in Fig. 11, while the reconstructed image is plotted in Fig. 12. The contrast of the reconstructed images and the resolution corresponding to the different apertures is computed and plotted in Table 2. The open circular aperture has improved contrast compared with the other modulated apertures, while the resolution is improved for the modulated apertures as shown from the precedent results. It is known early that the annular aperture will give an improvement in resolution compared with open circular aperture, while the contrast is decreased as expected.

**Table 2:** Resolution and contrast results for the reconstructed images for different apertures

Aperture	Resolution in reduced coo	Contrast = $\left( {I_{\max } - I_{\min } } \right)/\left( { I_{\max } + I_{\min } } \right)$
Polynomial 1	0.81	0.8359
Polynomial 2	0.76	0.8048 for COVID 19 image
0.9715 for Siemen’s star pattern		
Linear	0.86	0.8509
Quadratic	0.76	0.8298
Open circular	1.0	0.8772

## Conclusions

Firstly, the proposed models of polynomial apertures showed different PSF of improved resolution compared with the circular aperture. The second model of B/W polynomial aperture showed further improvement of resolution compared with the circular and linear apertures. These apertures are considered as amplitude filters where the phase is constant like the open circular aperture since the aperture phase is responsible on the aberration. The influence of the number of zones on the PSF is discussed showing resolution improvement for greater number of zones (*N* ≥ 5). It is shown that cut-off value = 0.7128 for *N* = 5 → 8 as compared with 0.8603 for *N* = 1 for linear aperture.

Secondly, the CTF is computed from the autocorrelation function corresponding to the polynomial apertures. The CTF corresponding to the second model is different from that corresponding to the autocorrelation of the first model and both are different from the CTF corresponding to the circular apertures. It is noted that the total band width for all apertures is two times the aperture diameter as expected from the autocorrelation of a finite object.

Finally, the reconstructed images obtained using the CSLM provided with the polynomial apertures are given where the original image is the Siemen’s star pattern.

## Data Availability

Not applicable.

## Abbreviations

FFT: Fast Fourier transform
PSF: Point spread function
NA: Numerical aperture
B/W: Black and white
CTF: Coherent transfer function
CSLM: Confocal scanning laser microscope
